# 4-Chloro-2-methyl-*N*-(2-methyl­phen­yl)­benzene­sulfonamide

**DOI:** 10.1107/S1600536809009623

**Published:** 2009-03-19

**Authors:** B. Thimme Gowda, Sabine Foro, P. G. Nirmala, Hiromitsu Terao, Hartmut Fuess

**Affiliations:** a Department of Chemistry, Mangalore University, Mangalagangotri 574 199, Mangalore, India; b Institute of Materials Science, Darmstadt University of Technology, Petersenstrasse 23, D-64287 Darmstadt, Germany; c Faculty of Integrated Arts and Sciences, Tokushima University, Minamijosanjima-cho, Tokushima 770-8502, Japan

## Abstract

In the crystal structure of the title compound, C_14_H_14_ClNO_2_S, the two aromatic rings are tilted relative to each other by 45.8 (1)°. In the crystal, inversion dimers linked by pairs of N—H⋯O hydrogen bonds occur.

## Related literature

For related structures, see: Gelbrich *et al.* (2007 ▶); Gowda *et al.* (2009**a* ▶,b* ▶); Perlovich *et al.* (2006 ▶).
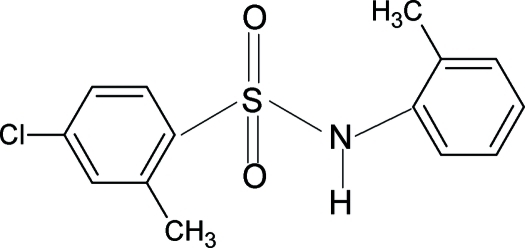


## Experimental

### 

#### Crystal data

C_14_H_14_ClNO_2_S
*M* *_r_* = 295.77
Triclinic, 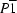

*a* = 8.1200 (8) Å
*b* = 8.1832 (8) Å
*c* = 10.985 (1) Å
α = 95.81 (1)°
β = 96.92 (1)°
γ = 106.82 (1)°
*V* = 686.46 (11) Å^3^
*Z* = 2
Cu *K*α radiation
μ = 3.86 mm^−1^
*T* = 299 K
0.50 × 0.48 × 0.18 mm

#### Data collection

Enraf–Nonius CAD-4 diffractometer
Absorption correction: ψ scan (North *et al.*, 1968 ▶) *T*_min_ = 0.228, *T*_max_ = 0.500
2572 measured reflections
2366 independent reflections
2194 reflections with *I* > 2σ(*I*)
*R*_int_ = 0.054
3 standard reflections frequency: 120 min intensity decay: 2.5%

#### Refinement

*R*[*F* ^2^ > 2σ(*F* ^2^)] = 0.072
*wR*(*F* ^2^) = 0.235
*S* = 1.10
2366 reflections
178 parameters
H atoms treated by a mixture of independent and constrained refinement
Δρ_max_ = 0.82 e Å^−3^
Δρ_min_ = −0.63 e Å^−3^

### 

Data collection: *CAD-4-PC* (Enraf–Nonius, 1996 ▶); cell refinement: *CAD-4-PC*; data reduction: *REDU4* (Stoe & Cie, 1987 ▶); program(s) used to solve structure: *SHELXS97* (Sheldrick, 2008 ▶); program(s) used to refine structure: *SHELXL97* (Sheldrick, 2008 ▶); molecular graphics: *PLATON* (Spek, 2009 ▶); software used to prepare material for publication: *SHELXL97*.

## Supplementary Material

Crystal structure: contains datablocks I, global. DOI: 10.1107/S1600536809009623/bt2900sup1.cif

Structure factors: contains datablocks I. DOI: 10.1107/S1600536809009623/bt2900Isup2.hkl

Additional supplementary materials:  crystallographic information; 3D view; checkCIF report

## Figures and Tables

**Table 1:** Hydrogen-bond geometry (Å, °)

*D*—H⋯*A*	*D*—H	H⋯*A*	*D*⋯*A*	*D*—H⋯*A*
N1—H1N⋯O2^i^	0.87 (4)	2.14 (4)	2.993 (4)	167 (3)

Symmetry code: (i) 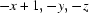.

**Fig. 1.**
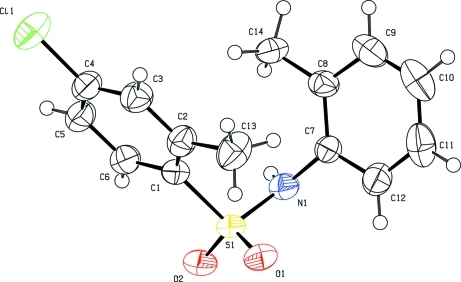
Molecular structure of the title compound showing the atom labeling scheme. The displacement ellipsoids are drawn at the 50% probability level. The H atoms are represented as small spheres of arbitrary radii.

**Fig. 2.**
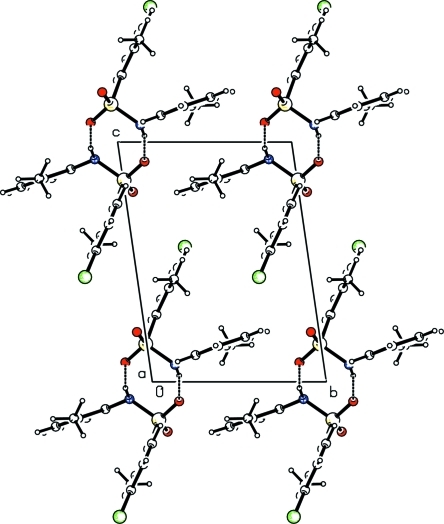
Molecular packing of of the title compound with hydrogen bonding shown as dashed lines.

## supplementary crystallographic information

### Comment

In the present work, as part of a study of substituent effects on the structures of *N*-(aryl)-arylsulfonamides, the structure of 4-chloro-2-methyl-*N*-(2-methylphenyl)benzenesulfonamide has been determined (Gowda *et al.* 2009*a,b*). The conformations of the N—C bonds in the C—SO_2_—NH—C segment have *trans* and *gauche* torsion angles with the S=O bonds (Fig. 1). The molecule is bent at the S atom with the C—SO_2_—NH—C torsion angle of 73.0 (2). The *ortho*-methyl group in the sulfonyl benzene ring is oriented away from the S=O bonds and so also the *ortho*-methyl group in the anilino benzene ring from the N—H bond. The two benzene rings are tilted relative to each other by 45.8 (1)°, compared with the values of 86.6 (2)° (molecule 1) and 83.0 (2)° (molecule 2), in the two independent molecules of 4-chloro-2-methyl- *N*-(phenyl)benzenesulfonamide  (Gowda *et al.*, 2009*a*). The other bond parameters in the title compound are similar to those observed in   2,4-dimethyl-*N*-(phenyl)benzenesulfonamide (Gowda *et al.*, 2009*b*) and other aryl sulfonamides (Perlovich *et al.*, 2006; Gelbrich *et al.*, 2007). The crystal packing of the molecules is characterized by N—H···O(S) hydrogen bonds (Table 1, Fig.2) .

### Experimental

A solution of *m*-chlorotoluene (10 cc) in chloroform (40 cc) was treated dropwise with chlorosulfonic acid (25 cc) at 0 ° C. After the initial evolution of hydrogen chloride subsided, the reaction mixture was brought to room temperature and poured into crushed ice in a beaker. The chloroform layer was separated, washed with cold water and allowed to evaporate slowly. The residual 4-chloro-2-methylbenzenesulfonylchloride was treated with *o*-toluidine in the stoichiometric ratio and boiled for ten minutes. The reaction mixture was then cooled to room temperature and added to ice cold water (100 cc). The resultant solid 4-chloro-2-methyl-*N*- (2-methylphenyl)benzenesulfonamide was filtered under suction and washed thoroughly with cold water. It was then recrystallized to constant melting point from dilute ethanol. The purity of the compound was checked and characterized by recording its infrared and NMR spectra. The single crystals used in X-ray diffraction studies were grown in ethanolic solution by slow evaporation at room temperature.

### Refinement

The H atom of the NH group was located in a diffrerence map and its position was refined.  The other H atoms were positioned with idealized geometry using a riding model [C—H = 0.93–0.96 Å]. All H atoms were refined with isotropic displacement parameters (set to 1.2 times of the *U*_eq_ of the parent atom) or *U*_iso_(H) = 1.5 *U*_eq_ for methyl groups.

To improve considerably the values of R1, wR2, and GOOF these   four reflections (-6 1 0, -2 3 5, 1 5 1, -2 -2 9) were omitted from the refinement.

### Crystal data

**Table:**

C_14_H_14_ClNO_2_S	*Z* = 2
*M**_r_* = 295.77	*F*(000) = 308
Triclinic, *P*1	*D*_x_ = 1.431 Mg m^−^^3^
Hall symbol: -P 1	Cu *K*α radiation, λ = 1.54180 Å
*a* = 8.1200 (8) Å	Cell parameters from 25 reflections
*b* = 8.1832 (8) Å	θ = 5.7–25.2°
*c* = 10.985 (1) Å	µ = 3.86 mm^−^^1^
α = 95.81 (1)°	*T* = 299 K
β = 96.92 (1)°	Prism, colourless
γ = 106.82 (1)°	0.50 × 0.48 × 0.18 mm
*V* = 686.46 (11) Å^3^	

### Data collection

**Table:**

Enraf–Nonius CAD-4 diffractometer	2194 reflections with *I* > 2σ(*I*)
Radiation source: fine-focus sealed tube	*R*_int_ = 0.054
graphite	θ_max_ = 66.9°, θ_min_ = 4.1°
ω/2θ scans	*h* = −9→1
Absorption correction: psi-scan (North *et al.*, 1968)	*k* = −9→9
*T*_min_ = 0.228, *T*_max_ = 0.500	*l* = −13→13
2572 measured reflections	3 standard reflections every 120 min
2366 independent reflections	intensity decay: 2.5%

### Refinement

**Table:**

Refinement on *F*^2^	Secondary atom site location: difference Fourier map
Least-squares matrix: full	Hydrogen site location: inferred from neighbouring sites
*R*[*F*^2^ > 2σ(*F*^2^)] = 0.072	H atoms treated by a mixture of independent and constrained refinement
*wR*(*F*^2^) = 0.235	*w* = 1/[σ^2^(*F*_o_^2^) + (0.1867*P*)^2^ + 0.2192*P*] where *P* = (*F*_o_^2^ + 2*F*_c_^2^)/3
*S* = 1.10	(Δ/σ)_max_ = 0.003
2366 reflections	Δρ_max_ = 0.82 e Å^−^^3^
178 parameters	Δρ_min_ = −0.63 e Å^−^^3^
0 restraints	Extinction correction: *SHELXL97* (Sheldrick, 2008), Fc^*^=kFc[1+0.001xFc^2^λ^3^/sin(2θ)]^-1/4^
Primary atom site location: structure-invariant direct methods	Extinction coefficient: 0.020 (4)

### Special details

**Table:**

Geometry. All e.s.d.'s (except the e.s.d. in the dihedral angle between two l.s. planes) are estimated using the full covariance matrix. The cell e.s.d.'s are taken into account individually in the estimation of e.s.d.'s in distances, angles and torsion angles; correlations between e.s.d.'s in cell parameters are only used when they are defined by crystal symmetry. An approximate (isotropic) treatment of cell e.s.d.'s is used for estimating e.s.d.'s involving l.s. planes.
Refinement. Refinement of *F*^2^ against ALL reflections. The weighted *R*-factor *wR* and goodness of fit *S* are based on *F*^2^, conventional *R*-factors *R* are based on *F*, with *F* set to zero for negative *F*^2^. The threshold expression of *F*^2^ > σ(*F*^2^) is used only for calculating *R*-factors(gt) *etc*. and is not relevant to the choice of reflections for refinement. *R*-factors based on *F*^2^ are statistically about twice as large as those based on *F*, and *R*- factors based on ALL data will be even larger.

### Fractional atomic coordinates and isotropic or equivalent isotropic displacement parameters (Å^2^)

**Table:**

	*x*	*y*	*z*	*U*_iso_*/*U*_eq_	
Cl1	0.35649 (13)	0.29943 (14)	0.56281 (9)	0.0698 (5)	
S1	0.70711 (9)	−0.00610 (8)	0.15793 (6)	0.0388 (4)	
O1	0.8587 (3)	−0.0468 (3)	0.2078 (2)	0.0511 (6)	
O2	0.5669 (3)	−0.1383 (3)	0.0812 (2)	0.0518 (7)	
N1	0.7667 (3)	0.1454 (3)	0.0718 (2)	0.0408 (6)	
H1N	0.678 (5)	0.160 (5)	0.026 (3)	0.049*	
C1	0.6176 (4)	0.0795 (4)	0.2802 (3)	0.0381 (7)	
C2	0.7184 (4)	0.1738 (4)	0.3914 (3)	0.0446 (8)	
C3	0.6322 (5)	0.2414 (4)	0.4757 (3)	0.0492 (8)	
H3	0.6954	0.3075	0.5496	0.059*	
C4	0.4541 (5)	0.2123 (4)	0.4518 (3)	0.0471 (8)	
C5	0.3557 (4)	0.1194 (4)	0.3436 (3)	0.0483 (8)	
H5	0.2361	0.1015	0.3284	0.058*	
C6	0.4388 (4)	0.0529 (4)	0.2572 (3)	0.0445 (7)	
H6	0.3743	−0.0105	0.1828	0.053*	
C7	0.9159 (4)	0.2918 (4)	0.1137 (2)	0.0370 (7)	
C8	0.8982 (4)	0.4545 (4)	0.1516 (3)	0.0417 (7)	
C9	1.0502 (6)	0.5921 (4)	0.1866 (3)	0.0580 (9)	
H9	1.0420	0.7016	0.2100	0.070*	
C10	1.2117 (6)	0.5710 (5)	0.1876 (4)	0.0654 (11)	
H10	1.3113	0.6648	0.2132	0.078*	
C11	1.2262 (5)	0.4090 (6)	0.1501 (4)	0.0655 (11)	
H11	1.3357	0.3943	0.1511	0.079*	
C12	1.0791 (5)	0.2706 (5)	0.1118 (3)	0.0516 (8)	
H12	1.0889	0.1629	0.0846	0.062*	
C13	0.9139 (5)	0.2097 (6)	0.4271 (3)	0.0607 (10)	
H13A	0.9401	0.1025	0.4258	0.073*	
H13B	0.9513	0.2741	0.5088	0.073*	
H13C	0.9736	0.2753	0.3691	0.073*	
C14	0.7233 (5)	0.4810 (4)	0.1522 (4)	0.0570 (10)	
H14A	0.6460	0.4174	0.0787	0.068*	
H14B	0.7352	0.6015	0.1546	0.068*	
H14C	0.6767	0.4410	0.2239	0.068*	

### Atomic displacement parameters (Å^2^)

**Table:**

	*U*^11^	*U*^22^	*U*^33^	*U*^12^	*U*^13^	*U*^23^
Cl1	0.0723 (8)	0.0846 (8)	0.0629 (7)	0.0384 (6)	0.0258 (5)	−0.0018 (5)
S1	0.0430 (6)	0.0280 (5)	0.0466 (5)	0.0131 (3)	0.0107 (3)	−0.0002 (3)
O1	0.0554 (14)	0.0426 (12)	0.0650 (14)	0.0270 (10)	0.0156 (11)	0.0088 (10)
O2	0.0570 (15)	0.0327 (11)	0.0614 (14)	0.0106 (10)	0.0117 (11)	−0.0058 (9)
N1	0.0440 (15)	0.0362 (13)	0.0422 (13)	0.0137 (11)	0.0079 (10)	−0.0001 (10)
C1	0.0431 (17)	0.0308 (14)	0.0427 (15)	0.0129 (11)	0.0114 (12)	0.0052 (11)
C2	0.0422 (18)	0.0510 (17)	0.0449 (16)	0.0191 (13)	0.0091 (13)	0.0103 (13)
C3	0.052 (2)	0.0490 (18)	0.0446 (16)	0.0155 (14)	0.0073 (14)	−0.0003 (13)
C4	0.053 (2)	0.0497 (18)	0.0484 (17)	0.0255 (15)	0.0195 (14)	0.0119 (14)
C5	0.0429 (18)	0.0519 (18)	0.0543 (18)	0.0202 (14)	0.0119 (14)	0.0052 (14)
C6	0.0441 (18)	0.0426 (16)	0.0473 (16)	0.0147 (13)	0.0081 (13)	0.0037 (12)
C7	0.0427 (16)	0.0365 (14)	0.0328 (13)	0.0119 (12)	0.0113 (11)	0.0043 (10)
C8	0.058 (2)	0.0339 (14)	0.0338 (14)	0.0128 (13)	0.0117 (12)	0.0031 (11)
C9	0.075 (3)	0.0388 (17)	0.0516 (18)	0.0032 (15)	0.0113 (16)	0.0070 (13)
C10	0.059 (2)	0.058 (2)	0.062 (2)	−0.0077 (17)	0.0005 (17)	0.0193 (17)
C11	0.041 (2)	0.082 (3)	0.073 (2)	0.0107 (18)	0.0111 (16)	0.032 (2)
C12	0.049 (2)	0.0553 (19)	0.0581 (19)	0.0217 (15)	0.0186 (15)	0.0146 (15)
C13	0.043 (2)	0.080 (3)	0.0544 (19)	0.0199 (17)	−0.0005 (15)	−0.0072 (17)
C14	0.074 (2)	0.0400 (17)	0.069 (2)	0.0274 (17)	0.0329 (19)	0.0081 (15)

### Geometric parameters (Å, °)

**Table:**

Cl1—C4	1.734 (3)	C7—C12	1.388 (5)
S1—O1	1.427 (2)	C7—C8	1.407 (4)
S1—O2	1.431 (2)	C8—C9	1.390 (5)
S1—N1	1.633 (3)	C8—C14	1.498 (5)
S1—C1	1.778 (3)	C9—C10	1.370 (6)
N1—C7	1.424 (4)	C9—H9	0.9300
N1—H1N	0.87 (4)	C10—C11	1.391 (7)
C1—C6	1.392 (5)	C10—H10	0.9300
C1—C2	1.400 (4)	C11—C12	1.373 (5)
C2—C3	1.389 (5)	C11—H11	0.9300
C2—C13	1.522 (5)	C12—H12	0.9300
C3—C4	1.383 (5)	C13—H13A	0.9600
C3—H3	0.9300	C13—H13B	0.9600
C4—C5	1.366 (5)	C13—H13C	0.9600
C5—C6	1.383 (5)	C14—H14A	0.9600
C5—H5	0.9300	C14—H14B	0.9600
C6—H6	0.9300	C14—H14C	0.9600
			
O1—S1—O2	119.17 (14)	C8—C7—N1	121.0 (3)
O1—S1—N1	108.06 (14)	C9—C8—C7	117.4 (3)
O2—S1—N1	105.21 (14)	C9—C8—C14	120.8 (3)
O1—S1—C1	109.71 (14)	C7—C8—C14	121.9 (3)
O2—S1—C1	106.93 (14)	C10—C9—C8	121.9 (3)
N1—S1—C1	107.12 (13)	C10—C9—H9	119.1
C7—N1—S1	121.1 (2)	C8—C9—H9	119.1
C7—N1—H1N	118 (2)	C9—C10—C11	119.8 (3)
S1—N1—H1N	112 (3)	C9—C10—H10	120.1
C6—C1—C2	121.0 (3)	C11—C10—H10	120.1
C6—C1—S1	115.7 (2)	C12—C11—C10	120.1 (4)
C2—C1—S1	123.3 (2)	C12—C11—H11	119.9
C3—C2—C1	116.9 (3)	C10—C11—H11	119.9
C3—C2—C13	117.4 (3)	C11—C12—C7	119.8 (3)
C1—C2—C13	125.6 (3)	C11—C12—H12	120.1
C4—C3—C2	121.3 (3)	C7—C12—H12	120.1
C4—C3—H3	119.4	C2—C13—H13A	109.5
C2—C3—H3	119.4	C2—C13—H13B	109.5
C5—C4—C3	121.7 (3)	H13A—C13—H13B	109.5
C5—C4—Cl1	120.0 (3)	C2—C13—H13C	109.5
C3—C4—Cl1	118.3 (3)	H13A—C13—H13C	109.5
C4—C5—C6	118.1 (3)	H13B—C13—H13C	109.5
C4—C5—H5	120.9	C8—C14—H14A	109.5
C6—C5—H5	120.9	C8—C14—H14B	109.5
C5—C6—C1	120.9 (3)	H14A—C14—H14B	109.5
C5—C6—H6	119.6	C8—C14—H14C	109.5
C1—C6—H6	119.6	H14A—C14—H14C	109.5
C12—C7—C8	121.0 (3)	H14B—C14—H14C	109.5
C12—C7—N1	118.0 (3)		
			
O1—S1—N1—C7	−45.5 (2)	Cl1—C4—C5—C6	179.7 (2)
O2—S1—N1—C7	−173.8 (2)	C4—C5—C6—C1	0.2 (5)
C1—S1—N1—C7	72.7 (2)	C2—C1—C6—C5	0.2 (5)
O1—S1—C1—C6	−152.0 (2)	S1—C1—C6—C5	−177.4 (2)
O2—S1—C1—C6	−21.4 (3)	S1—N1—C7—C12	75.9 (3)
N1—S1—C1—C6	90.9 (2)	S1—N1—C7—C8	−106.4 (3)
O1—S1—C1—C2	30.4 (3)	C12—C7—C8—C9	0.1 (4)
O2—S1—C1—C2	161.0 (3)	N1—C7—C8—C9	−177.5 (3)
N1—S1—C1—C2	−86.6 (3)	C12—C7—C8—C14	178.9 (3)
C6—C1—C2—C3	−1.1 (5)	N1—C7—C8—C14	1.3 (4)
S1—C1—C2—C3	176.3 (2)	C7—C8—C9—C10	−1.6 (5)
C6—C1—C2—C13	179.4 (3)	C14—C8—C9—C10	179.5 (3)
S1—C1—C2—C13	−3.1 (5)	C8—C9—C10—C11	1.5 (6)
C1—C2—C3—C4	1.8 (5)	C9—C10—C11—C12	0.3 (6)
C13—C2—C3—C4	−178.7 (3)	C10—C11—C12—C7	−1.8 (5)
C2—C3—C4—C5	−1.5 (5)	C8—C7—C12—C11	1.6 (5)
C2—C3—C4—Cl1	179.3 (2)	N1—C7—C12—C11	179.3 (3)
C3—C4—C5—C6	0.5 (5)		

### Hydrogen-bond geometry (Å, °)

**Table:**

*D*—H···*A*	*D*—H	H···*A*	*D*···*A*	*D*—H···*A*
N1—H1N···O2^i^	0.87 (4)	2.14 (4)	2.993 (4)	167 (3)

Symmetry codes: (i) −*x*+1, −*y*, −*z*.
